# The role of artificial intelligence in hypertension management

**DOI:** 10.1097/MNH.0000000000001157

**Published:** 2026-01-05

**Authors:** Mukesh Dherani, Siegfried K. Wagner, Eduard Shantsila

**Affiliations:** a Department of Primary Care and Mental Health, University of Liverpool; b NIHR Biomedical Research Centre, Moorfields Eye Hospital; c Institute of Ophthalmology, University College London, London, UK

**Keywords:** artificial intelligence, blood pressure, hypertension, machine learning

## Abstract

**Purpose of review:**

Hypertension remains a leading modifiable risk factor for cardiovascular and renal conditions and dementia. Given its rising global prevalence and economic burden, artificial intelligence offers promising solutions across the care continuum, from diagnosis to monitoring. This review highlights recent advances in artificial intelligence-driven diagnosis and monitoring, risk stratification, and predictive modelling of hypertension-related outcomes.

**Recent findings:**

Using artificial intelligence-based technologies, validated wearable cuffless monitors are developed, which use electrocardiography, heart sounds, and thoracic impedance data and provide continuous blood pressure (BP) monitoring. Artificial intelligence-generated algorithm have shown promising response to accurately predict BP. The Extreme Gradient Boost has consistently performed as the best algorithms. Additionally, these models have been used in predicting hypertension impact on cardiovascular, renal, and retinal conditions, and in predicting treatment strategies. Emerging applications of Large Language Models are being developed to provide personalized care based on individual patient characteristics.

**Summary:**

Artificial intelligence has the potential to transform hypertension management through improved diagnosis, monitoring, and personalized care and prediction of its systemic consequences. However, challenges of model validation, interpretability, generalizability, and ethics persist. Robust prospective trials and equitable implementation strategies can help realise the potential of artificial intelligence in improving hypertension outcomes.

## INTRODUCTION

Hypertension, a major global health burden with an estimated prevalence of 1.28 billion adults worldwide in 2023 [1], is a leading modifiable risk factor for cardiovascular disease, chronic kidney disease, and dementia and implicated in more than 10 million deaths annually [2]. Hypertension is underdiagnosed and despite the availability of effective and efficacious pharmacological and lifestyle interventions, only 21–26% of patients achieve recommended blood pressure (BP) targets [3]. There is an obvious differentially poor attainment in resource-limited settings. While 28.7% of treated individuals in high-income countries achieve sufficient BP control, the corresponding figure in low-income and middle-income countries is only 7.7% [3].

High prevalence and poor control of hypertension are disproportionately concentrated among socioeconomically disadvantaged groups and racial and ethnic minority populations, reflecting the influence of structural inequities and social determinants of health, including healthcare access, health literacy, and environmental exposures [2]. In the United Kingdom (UK), approximately 30% of those aged 18 or more are hypertensive, with more than 5.5 million individuals undiagnosed, more often in ethnic minority groups [4]. In the United States, BP control rates have declined since 2014, with the steepest reductions observed among non-Hispanic Black and Hispanic populations [5]. The economic burden is considerable, with annual costs estimated at £15.8 billion in the UK [6] and $417.9 billion in the United States between 2020 and 2021, encompassing both direct healthcare expenditures and indirect societal costs [7]. With demographic transitions toward an increasingly aged population, and the consequent anticipated rise in hypertension prevalence, there is a pressing need to develop and implement innovative strategies to enhance detection and optimize management.

We conducted a literature review of studies published between 2024 and 2025 to examine the role of artificial intelligence in the diagnosis and management of hypertension, as well as its broader clinical consequences. The review synthesizes current evidence on how artificial intelligence-based approaches including predictive algorithms, clinical decision support systems, and wearable technologies are being applied in hypertension care. We also assessed the key limitations of existing applications, particularly with respect to data quality, generalizability, and model transparency. Based on these findings, future directions aimed to support the development of equitable, effective, and clinically integrated artificial intelligence systems for hypertension management are proposed. 

**Box 1:**
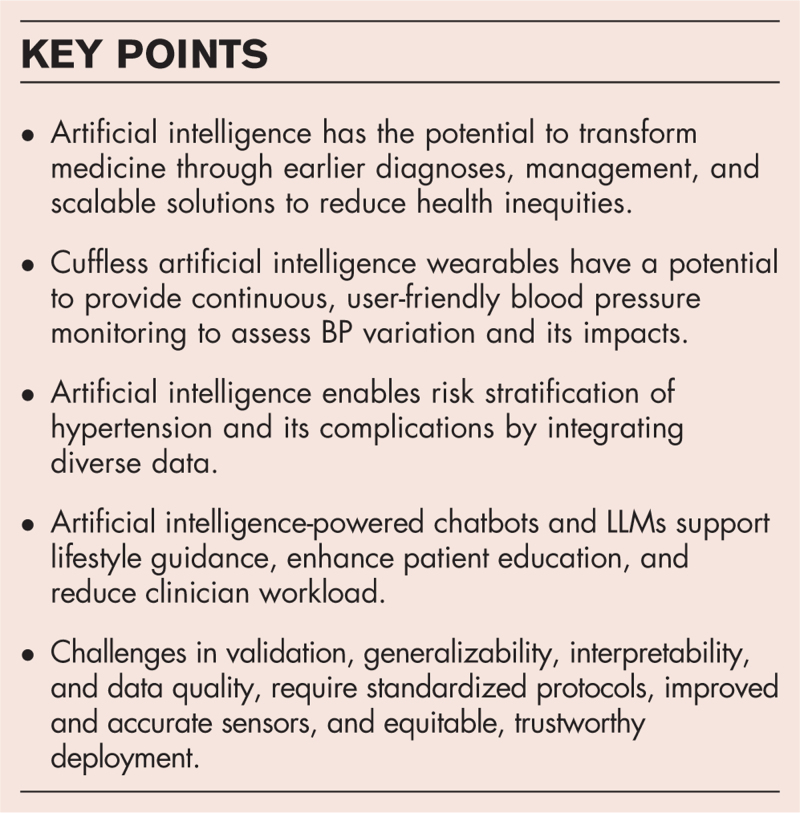
no caption available

## ARTIFICIAL INTELLIGENCE

Artificial intelligence has emerged as a transformative paradigm within medicine, with expanding applications in disease detection, risk stratification, therapeutic decision-making, and health system optimization. Its applications include imaging analysis for early disease detection and drug discovery, and clinical decision support systems for more informed decisions. Furthermore, artificial intelligence is used in patient monitoring to track vital signs in real-time, in public health to predict and manage disease outbreaks, and through natural language processing (NLP), to streamline administrative tasks and improve communication, ultimately making healthcare more efficient and effective.

Conceptually, artificial intelligence encompasses computational architectures capable of reasoning, adaptive learning, and autonomous decision-making, with recent advances driven predominantly by machine learning, and particularly deep learning methodologies [2]. The trajectory of machine learning development from early neural networks in the 1940s, through the perceptron in the 1950s, to the landmark achievement of IBM's Deep Blue defeating Garry Kasparov in 1997 has been enabled by exponential growth in computational capacity and data availability [8]. Artificial intelligence is increasingly embedded into clinical practice guidelines, decision-support frameworks, and medical education [9].

Machine learning-based models integrating clinical and biomarker data have achieved diagnostic accuracy matching or exceeding the accuracy of conventional methods in a number of clinical fields and artificial intelligence-enabled technologies offer scalable diagnostic capacity in resource-limited settings [10]. While conventional approaches to hypertension screening and case identification have proven insufficient to address the scale of the disease burden, emerging artificial intelligence technologies may provide a pathway toward precision medicine, with potential for earlier hypertension diagnosis, personalized therapeutic strategies, optimized healthcare delivery, and reduction of health inequities.

## DIAGNOSIS AND SCREENING USING WEARABLE BLOOD PRESSURE MONITORS

Diagnosis and monitoring of hypertension are key to successful detection and treatment of hypertension. Traditional office-based BP measurements offer limited insight as they provide a single cross-sectional estimate [11,12^▪^] and may lead to misdiagnosis due to white coat hypertension or masked hypertension. The ambulatory BP monitoring (ABPM), considered the gold standard [13^▪^] in BP assessment, is limited by its availability, patient discomfort due to frequent cuff inflations, sleep disturbances, and limited reproducibility at the individual level [12^▪^].

Wearable, cuffless technologies offer a noninvasive, continuous, and user-friendly alternative. Such devices can detect masked or white-coat hypertension and describe real-time BP variations, which is critical for early intervention and personalized care [14^▪▪^,^15^]. The core methods employed by wearable BP monitors include photoplethysmography (PPG), electrocardiography [16], and advanced sensor technologies [17] do not directly measure BP. They require increasingly utilized artificial intelligence algorithms to estimate SBP and DBP from physiological signals. For instance, the SimpleSense-BP platform uses electrocardiography, heart sounds, and thoracic impedance data processed through artificial intelligence to estimate BP across all hypertension stages, achieving mean absolute differences within 5 mmHg of reference values obtained using standard sphygmomanometer BP measurements [14^▪▪^]. Similarly, flexible electronics and sensor arrays enable skin-conformal, continuous monitoring with high sensitivity and comfort [15,18].

## RISK STRATIFICATION

Another important element of artificial intelligence use in hypertension is in prediction of hypertension and its possible adverse consequences (Table 1). The traditional risk scores rely on linear models and a limited number of health variables, which may not capture the complex, multifactorial nature of cardiovascular effects of hypertension. QRISK, a widely used risk scoring system in UK primary care for CVD prediction, including for decisions of starting BP-lowering treatment, was found to have inaccurate prediction across age groups [19]. Artificial intelligence models, in contrast, can process large-scale, multimodal datasets, including clinical, genetic, behavioural, and environmental data to identify a variety of patterns and phenotypes that inform more accurate risk prediction and management strategies [8,20]. Artificial intelligence-based models used in Norway study found age, BP, BMI, and family history can predict hypertension even with normal BP as baseline [21^▪^].

**Table 1:** Key studies of artificial intelligence-based approaches for hypertension

Author	Population	Input data	Outcomes	Models	Results
Sau *et al*. [27^▪▪^]	US cohorts (*n* = 189 539), UK Biobank (35 806)	ECG	Hypertension CV death Heart failure Myocardial infarction Ischaemic stroke Chronic kidney disease	AI-ECG Risk Estimation platform (AIRE)	Model was predictive of the tested outcomes
Cavero-Redondo *et al.*[33^▪^]	Three Spanish cohorts (*n* = 194)	Antihypertensive medications; patient characteristics including demographic and clinical data	Reduction in PWV using antihypertensive medication	Random forest	Drugs reduce PWV: ARBs: cholesterol ACEi: weight and glycated haemoglobin
Tran *et al.*[13^▪^]	Three UK cohorts (*n*: 923; 709; 1222)	Office BP, ABPM, and clinical, laboratory, and demographic data	Diagnosis of hypertension types	Scikit-learn; XGBoost	XGBoost was best performing model
Squirrell *et al.*[24^▪^]	UK Biobank (*n* = 51 778)	Colour fundus photography	CVD events	Five DL models, each an iteration of ResNet architecture	predicted CVD events
Mohammadi *et al.*[12^▪^]	US cohort (*n* = 2064)	Multiparameter monitoring in intensive care II	Cuffless BP monitoring	Hybrid DL models: CNN and BiLSTM	Cuff-less AI-based BP monitors provides more precise information
Yi *et al.*[35^▪^]	Chinese cohort tanking antihypertensive (*n* = 6282)	Participants from CRT were used with data on demographic and co-morbidities	BP response to BP-lowering drugs	Five ML models LR, RF, XGBoost, TabNet, and Catboost	XGBoost predicted better than other models
Schjerven *et al.*[21^▪^]	Norwegian cohort (HUNTS study data) [46] (*n* = 17 852)	Adults 20–85 years, with BP below hypertension threshold	Hypertension diagnosis over 11 years	Six ML models: XGBoost, Elastic regression, K-Nearest Neighbor, SVM, and Random Forest compared with logistic regression	XGBoost, Elastic regression and SVM performed better
Mizuno *et al.*[29^▪^]	Japanese cohort of pregnant women (*n* = 23 790)	Early pregnant ladies; mean gestation: 20.2 weeks; lifestyle factors	Early hypertension in pregnancy	Logistic regression, random forest, XGBoost	Questionnaire and SVP models predicted best HTN
Bisong *et al.*[23^▪▪^]	57 countries from 6 WHO regions (*n* = 184 674)	18–69 years, demographic, behavioural, clinical data	Prediction of hypertension status	LR, KNN; RF; XGBoost; Fully Connected Neural Network	Model performance varied from 59–84% precision
Leitner *et al.*[37^▪^]	USA hypertension cohort from single arm, nonrandomised trial (*n* = 141)	Hypertensive patients aged 18 and above who own smart phone	Reduction in BP in response to AI based lifestyle coaching	No model was used	Significant reduction in BP at 24 weeks
Schor *et al.*[30^▪^]	USA nulliparous women (*n* = 9124)	Low-risk women	Hypertension prediction from 20 weeks of gestation to 2 weeks postpartum	RF with recursive feature elimination	Models help predict HDP and may guide aspirin prescription in high-risk pregnancies
Mroz *et al.*[31^▪^]	US cohort (*n* = 350 008)	Adults, 18 and above with clinical and lab data	Hypertension control at 12 months	XGBoost	AUC to predict control: 0.756

ACEi, angiotensin-converting enzyme inhibitors; ARB, angiotensin receptor blocker; HDP, hypertensive disease of pregnancy; KNN, K-Nearest neighbours; LR, linear regression; PWV, pulse wave velocity; RF, random forest models; SVM, Support Vector Machines; XGBoost, Extreme Gradient Boosting.

Artificial intelligence is also being applied to stratify risk for adverse outcomes in patients with hypertension. For example, artificial intelligence models trained on electronic health records can forecast cardiovascular events, treatment response, and mortality risk with greater accuracy than conventional tools. In cardiac intensive care and postacute coronary syndrome settings, artificial intelligence-derived scores such as PRAISE predicted 1-year mortality and bleeding risk better than established clinical scores [22].

Moreover, artificial intelligence tools can help predict the onset of hypertension. machine learning-based algorithms such as support vector machines (SVM), random forests, and extreme gradient boosting (XGBoost) consistently outperform traditional statistical models in hypertension prediction. These models have achieved high predictive performance, with area under the ROC curve (AUROC) values ranging from 0.766 to 1.00 across diverse populations and datasets [20]. Machine learning has been used to integrate omics data with lifestyle and socioeconomic factors, enabling the identification of novel risk factors and subgroups within hypertensive populations [8,20,22]. A cross-sectional study conducted in 57 countries to assess the demographic, behavioural, clinical, and lab-based data found that global XGBoost was most accurate (68.5%), while regional accuracy was variable [23^▪▪^].

Deep learning models can also infer BP from retinal imaging, along with other key cardiovascular risk factors like age, smoking status, and BMI [24^▪^,^25^]. Such “AI-predicted BP” has been shown to be a significant predictor of future major cardiovascular events, such as myocardial infarctions and strokes [26]. Deep learning models have been trained to analyse the raw data from a standard 12-lead ECG and identify subtle patterns that indicate the presence of hypertension [27^▪▪^].

Early identification of preeclampsia can reduce the maternal and infant outcomes in pregnant women. The artificial intelligence has been effectively used where the principles of frequent, out-of-office monitoring are required. Machine learning models use home BP monitoring data, which provides a similar longitudinal view as ABPM, to predict the onset of hypertensive disorders of pregnancy [28]. The artificial intelligence-driven multiomics approaches (e.g. genomics, metabolomics, transcriptomics) have achieved more precise performance (accuracy: 94.6%; AUC: 0.99) [9]. A study in Japan found that comprehensive lifestyle data can predict hypertension in pregnancy (AUC: 0.93) [29^▪^]. Another study using data used from low-risk nulliparous pregnant women in the United States showed that random forest model accurately predicted pregnancy related hypertension (AUC: 0.73) [30^▪^].

## TREATMENT OPTIMIZATION

Improved BP control is vital to mitigating morbidity risk and earlier BP control results in lower morbidity and mortality [16,31^▪^]. Application of artificial intelligence in hypertension management may allow a more personalized approach to treatment [32]. Instead of relying on traditional one-size-fits-all guidelines and a trial-and-error method for prescribing BP-lowering medications to achieve the goal, Machine learning models have been developed for understanding individual-level treatment response and consequently recommendations for optimal therapeutic strategy from the outset. Furthermore, machine learning models can help select medications to target specific underlying cause, such as arterial stiffness, and recommend specific medications [33^▪^].

There are still limited data on the ability of artificial intelligence-driven approaches to provide robust decision support tools for the selection of medications and medication dosage to achieve BP reduction safely and effectively. Possible variables that may help include current antihypertensive medications, baseline BP, age, kidney function, and ethnic background [34]. Such models allow clinicians to identify high-risk patients who may require more intensive or specialized management strategies if the standard treatment was not adequate. A prospective data analysis from China involving 6282 hypertension patients used 12 demographic and clinical features to predict the medication response to BP [35^▪^]. A retrospective data analysis of repository data from electronic health records of 350 008 (10 564 174 data points) to assess the control of BP at 12 months showed moderate prediction of BP control (AUC: 0.756) [31^▪^].

## PERSONALIZED CARE: THERAPEUTICS, CHATBOTS, AND LARGE LANGUAGE MODELS

Artificial intelligence-powered chatbots are being developed and studied to provide scalable, continuous support to patients with hypertension, bridging the gap between clinical visits [36]. The technology behind modern chatbots, including Large Language Models (LLMs) like ChatGPT, is being explored for its potential to make these interactions more sophisticated and personalized [8]. In a single-arm nonrandomized trial, a digital platform offering tailored advice on diet, exercise, and other lifestyle factors led to a significant reduction in BP among adults with hypertension [37^▪^].

The LLMs can retrieve, summarize, and synthesize critical data from unstructured sources like electronic health records and clinical notes. This can save clinicians significant time, reduce administrative burden, and help in making more informed decisions by presenting the most relevant patient information concisely [38]. LLMs can also be used to generate clear, easy-to-understand educational materials for patients, helping them to better understand their condition and treatment plan [8].

## LIMITATIONS OF ARTIFICIAL INTELLIGENCE IN HYPERTENSION MANAGEMENT

Artificial intelligence has vast potential in diagnosing and managing hypertension. Artificial intelligence can use novel digital interventions, such as promoting patient awareness, self-monitoring, healthy behaviours, and medication compliance [34,39–41]. However, its translation into routine clinical practice is hampered by critical issues related to validation, implementation, data quality, and generalizability, particularly when models trained on one population are applied to another [42]. Overfitting, data leakage, lack of interpretability, and potential biases in training data also limit clinical adoption. Efforts to develop explainable artificial intelligence (XAI) and standardized validation protocols are ongoing to address these issues [8,20]. They should also consider the issue of data poverty with certain populations being poorly represented in current artificial intelligence research and data science, and consequently limiting the generalizability of artificial intelligence tools [43].

Many artificial intelligence applications are still in the ‘proof-of-concept’ stage. Although they show promise in research settings, they lack the large-scale, prospective clinical trials needed to prove their efficacy, safety, and utility in real-world clinical environments. There is a critical need to bridge the gap between development and real-world implementation [2,5,34]. Clinicians are often hesitant to trust a prediction or recommendation if they cannot understand the underlying reasoning. Some advocate that trustworthy artificial intelligence tools should ideally offer an explanation that a healthcare professional can evaluate and trust, while others consider this less essential [44].

Wearable artificial intelligence-driven BP monitors face several challenges including motion artefacts, sensor placement variability, environmental interference (e.g., light, temperature), and the need for frequent calibration against cuff-based devices [16]. Many models struggle with generalizability across diverse populations due to physiological variability and limited training datasets [16,18]. The European Society of Hypertension (ESH) has stated the need for further validation of cuffless monitors, before these are used for hypertension diagnosis [45].

Future developments need standardized validation protocols, improved sensors, and hybrid artificial intelligence models that combine spatial and temporal signal features [45]. The integration of anomaly detection frameworks can enhance the identification of clinically relevant deviations in BP trends, supporting early diagnosis and intervention [17]. Additionally, ethical considerations, data privacy and confidentiality, and interoperability with electronic health records must be addressed to ensure scalable, secure, and equitable deployment of health technologies [8,17,22]. The future artificial intelligence model need to be generalizable and have taken into account sociodemographic and ethnic group differences (Table 1). They are required to follow the developments in understanding the mechanisms of hypertension effects on health, impacts of different BP metrices (e.g. SBP vs. DBP, central BP vs. peripheral BP, diurnal BP variations and responses of BP to physical and mental activities and stressors) and the advances in sensors used for BP recordings.

## CONCLUSION

Artificial intelligence offers powerful tools for enhancing hypertension diagnosis, risk stratification, and management. Their ability to integrate diverse data sources and adapt to individual patient profiles marks them as powerful potential enablers of accurate cardiovascular care. Most of the studies conducted have used datasets available from ongoing studies or repositories making it difficult to generalize to other populations. Robust clinical trials or prospective studies are needed to develop the models that use integrated data from diverse populations and input sources. This includes combining physiological signals (ECG, PPG), imaging data (including retinal photography), electronic health records, genomics, and lifestyle data to create a holistic and highly personalized risk profile for each patient. This could lead to more accurate predictions of hypertension, cardiovascular events, and treatment response. However, rigorous validation, regular calibration, meticulous transparency, and ethical oversight are essential to ensure equitable implementation.

## Acknowledgements

*None*.

### Financial support and sponsorship

*None.*

### Conflicts of interest

*There ae no conflicts of interest.*

## REFERENCES AND RECOMMENDED READING

Papers of particular interest, published within the annual period of review, have been highlighted as:

▪ of special interest
▪▪ of outstanding interest
